# Rationalizing manifold CSR dimensions-and-aspects towards sustainable business performance of small and medium-sized enterprises: Findings from pre-hoc and post-hoc analysis

**DOI:** 10.1371/journal.pone.0353992

**Published:** 2026-09-15

**Authors:** Ge Zhang, Gazi Md. Shakhawat Hossain, Jing Zhang, Mingxing Li, Md. Shahinur Rahman, Hafsa Binta Firdaus

**Affiliations:** 1 School of Economics and Management, Qinghai Minzu University, Xining, P. R. China; 2 Department of Management Studies, Faculty of Business Studies, University of Barishal, Kornokathi, Barishal, Bangladesh; 3 School of Management, Huazhong University of Science and Technology, Wuhan, P. R. China; 4 School of Management, Jiangsu University, Zhenjiang, Jiangsu, P.R. China; 5 Department of Marketing, Faculty of Business Studies, University of Barishal, Kornokathi, Barishal, Bangladesh; 6 Faculty of Business Studies, Port City International University, Chattogram, Bangladesh; Liaoning Normal University, CHINA

## Abstract

Despite increasing global focus on sustainability, small and medium-sized enterprises (SMEs) in developing countries remain understated in corporate social responsibility (CSR) research. Existing studies mainly emphasize large corporations, leaving a gap in understanding CSR’s role in SME sustainability. This study explores the strategic role of CSR in driving sustainable business performance (SBP) among SMEs in Bangladesh. While CSR is often treated as compliance, this research positions it as a proactive strategy for long-term sustainability. Using stakeholder theory (ST), the study combines pre-hoc and post-hoc analyses to examine CSR’s impact. A quantitative research design integrates Partial Least Squares Structural Equation Modeling (PLS-SEM) with Importance-Performance Map Analysis (IPMA). Data were collected through structured interviews with 350 SME stakeholders in the manufacturing sector. Results show a strong positive relationship between CSR dimensions and SBP. IPMA findings highlight the significance of multi-stakeholder communication in shaping CSR priorities. This study offers practical insights for managers, policymakers, and practitioners seeking to embed CSR in SME strategies. It calls for structured policies, financial support, and regulatory incentives to promote CSR adoption. The findings extend existing theory by linking CSR with sustainability through a stakeholder lens in resource-constrained settings. Methodologically, the study contributes by applying PLS-SEM and IPMA to assess CSR’s strategic influence. Moreover, the study findings offer novel insights into CSR’s strategic role in SME sustainability within developing economies.

## 1 Introduction

In recent years, corporate social responsibility (CSR) has been an important part of how businesses achieve sustainability. It no longer exists as an isolated function but as a key driver of long-term strategic performance [1]. Businesses increasingly view CSR as central to stakeholder engagement, resilience, and competitive advantage [2,3]. Global trends show a shift from profit-driven models to more ethically grounded business systems. These trends reflect growing concerns about environmental degradation, labor rights, and social inequality [4,5]. CSR’s role has shifted beyond philanthropy into a value-based approach grounded in accountability and transparency. Organizations now face a social contract that requires them to balance profit-making with societal obligations. Companies engaging meaningfully with CSR often experience enhanced reputation, stakeholder loyalty, and business continuity [6]. Yet, while CSR’s outcomes are well discussed, its internal mechanisms remain debated. Particularly in SMEs, the processes that align CSR activities with sustainable business performance (SBP) are still unclear. This underscores the need for focused and strategic CSR research in emerging markets.

The United Nations’ Sustainable Development Goals (SDGs) have further legitimized CSR as a tool for development. Companies are urged to adopt CSR practices that address social, economic, and environmental challenges simultaneously. Firms that embrace CSR are often more adaptive, innovative, and prepared for future uncertainties [7]. Empirical studies increasingly confirm positive relationships between CSR and corporate financial performance [8,9]. However, such findings offer limited insights into the mechanisms behind this association. Managers often remain uncertain about how to operationalize CSR within firm strategies. This ambiguity limits CSR’s transformative power, especially in contexts where resources are scarce. In small and medium-sized enterprises (SMEs), CSR implementation is often informal and reactive. These firms lack structured frameworks and decision-making tools to leverage CSR for performance gains. Understanding how CSR dimensions influence business outcomes is thus both theoretically and practically important. This study aims to address these gaps by examining CSR through a multi-dimensional and stakeholder-based lens.

Despite their critical economic contributions, SMEs remain underrepresented in CSR research. Most literature prioritizes large multinational corporations (MNCs) due to their global influence and reporting structures. Yet, SMEs account for over 90% of businesses globally and play a central role in innovation and employment [10,11]. In Bangladesh, SMEs drive local economies but face barriers to CSR adoption. These include financial limitations, lack of managerial expertise, and ambiguous regulatory frameworks [12,13]. CSR is often seen as a legal obligation, not a strategic investment. Consequently, SME engagement in CSR remains shallow and inconsistent. Few SMEs align CSR with performance goals or stakeholder needs. Moreover, the literature rarely considers the multi-stakeholder environment in which these firms operate. This has created a substantial gap in CSR research. Addressing this gap requires exploring both internal and external CSR elements in SMEs. Our study responds by integrating strategic and communication-based perspectives into CSR analysis.

CSR practice in Bangladesh is largely shaped by philanthropic tradition and regulatory minimalism. Many SMEs equate CSR with charitable donations rather than embedding it into business strategy [5,14]. Larger firms have begun aligning CSR with SDG objectives, but SMEs continue to lag [15]. Reasons include poor awareness, weak enforcement, and financial constraints [16]. The absence of structured governance allows short-term goals to override sustainable development principles. SMEs frequently avoid CSR investments to maintain profitability. These practices undermine long-term competitiveness and social legitimacy. The need for clear, enforceable CSR frameworks in Bangladesh is critical. Moreover, integrating CSR into business models can help SMEs improve resilience and stakeholder engagement. Limited CSR research in the SME context further justifies our study. Investigating how internal factors (e.g., employee welfare) and external factors (e.g., environmental compliance) impact performance is essential. This leads to our central inquiry, which guides the design and scope of this research. The central research question is: how do manifold dimensions and specific aspects of Corporate Social Responsibility (CSR) activities influence the sustainable business performance of small and medium-sized enterprises (SMEs), based on stakeholder-focused analysis using both pre-hoc and post-hoc methods?

Moreover, this study addresses the question through a communication-centered stakeholder approach. It evaluates how internal CSR (such as ethical governance and employee welfare) and external CSR (like community involvement and regulatory compliance) contribute to SBP. Additionally, this research identifies priority CSR dimensions using Importance-Performance Map Analysis (IPMA). By combining theoretical modeling with empirical testing, the study seeks to uncover actionable insights. It applies Partial Least Squares Structural Equation Modeling (PLS-SEM) for pre-hoc analysis and IPMA for post-hoc prioritization. This dual-method design ensures robustness and contextual relevance. Importantly, the study responds to calls for deeper stakeholder engagement in CSR research. It also brings attention to the implementation gap between CSR awareness and actual practices within SMEs. Our approach offers both explanatory and practical value for SME development and CSR policy formulation.

CSR must be understood as both a strategic and relational practice. Traditional CSR research focuses on financial performance and omits relational dynamics. Stakeholder theory provides a valuable framework to assess CSR’s effectiveness. Freeman’s model views firms as networks of relationships, each with unique interests and power dynamics. This aligns with Carroll’s CSR pyramid, which outlines four tiers of business responsibility—economic, legal, ethical, and philanthropic. When integrated, these models offer a comprehensive framework for evaluating CSR in SMEs. They allow researchers to examine how various CSR aspects influence internal and external stakeholder outcomes. This study uses this combined framework to assess CSR’s impact on SBP. It does so through a multi-stakeholder lens, capturing the perspectives of employees, customers, suppliers, and regulators. This adds a relational dimension to the analysis, enriching our understanding of CSR implementation. It also highlights the communication strategies firms use to engage stakeholders. These insights are essential to designing CSR models that support both accountability and growth.

In Bangladesh, many SMEs operate under volatile conditions with limited institutional support. As a result, more than 70% fail within five years [17,18]. Weak managerial capacity, poor planning, and undefined CSR practices often contribute to this failure. Yet, SMEs are pivotal to the country’s economic growth and social stability. Strategic CSR adoption can enhance SME resilience and sustainability. Our study analyzes both internal CSR (employee training, fair wages) and external CSR (community support, environmental management). We investigate how these practices link to firm-level performance metrics. Using both pre-hoc theoretical modeling and post-hoc performance mapping strengthens our findings. The research uncovers high-impact CSR areas where SMEs can invest resources efficiently. By identifying these leverage points, we offer actionable guidance. The study contributes to a growing literature advocating for data-driven, stakeholder-oriented CSR in developing economies. It also provides a foundation for future research into CSR’s role in firm strategy and performance.

This paper makes three core contributions to CSR and SME literature. First, it bridges the gap between Carroll’s CSR theory and Freeman’s stakeholder framework in SME settings. This integration enables a richer theoretical understanding of CSR’s strategic relevance. Second, it applies a novel dual-method approach using PLS-SEM and IPMA to analyze stakeholder-linked CSR performance. These tools identify which CSR dimensions most influence SBP in SMEs. Third, it delivers practical recommendations tailored to SMEs in resource-constrained environments. These include designing structured CSR strategies, aligning with SDGs, and fostering multi-stakeholder communication. The findings offer policy implications for regulators aiming to strengthen CSR frameworks in developing countries. They also provide managerial insights for SME owners seeking competitive and sustainable growth. Ultimately, this study enriches academic discourse by providing theoretical, empirical, and methodological advances. It also lays a strong foundation for future inquiry into CSR’s strategic role in emerging-market SMEs. The next sections present the literature review, methods, findings, and implications for practice and policy.

## 2 Literature review and hypothesis development

### 2.1 Underpinning theories

Stakeholder Theory (ST), first introduced by Freeman [19], emphasizes that firms must serve more than just shareholders. It proposes that sustainable success depends on balancing the interests of all stakeholder groups. These include internal stakeholders such as employees and managers and external ones, including customers, suppliers, communities, and regulators [19,20]. Rather than focusing solely on profit, ST suggests that businesses should foster long-term relationships and mutual value creation. Organizations that engage stakeholders meaningfully build trust, legitimacy, and organizational resilience [21]. In the context of CSR, ST provides a compelling rationale for integrating ethical, social, and environmental considerations into firm strategy. CSR is no longer viewed as a regulatory formality but as a tool for stakeholder alignment and sustainable advantage. This theory is particularly relevant for small and medium-sized enterprises (SMEs), where close-knit stakeholder relationships are common. ST thus forms the core theoretical lens for this study’s examination of CSR’s role in sustainable business performance (SBP).

CSR, viewed through the stakeholder lens, supports both relational and strategic business goals. Internally, CSR practices such as fair labor policies, workforce training, and ethical leadership build employee morale and productivity [22]. Externally, activities like transparent communication, customer care, and community engagement strengthen brand loyalty and stakeholder trust [23]. SMEs are uniquely positioned to benefit from such initiatives due to their local focus and relational proximity [11]. By incorporating stakeholder feedback into CSR strategies, SMEs can better navigate dynamic market conditions. Stakeholder Theory highlights how these CSR actions contribute to reputational capital, legitimacy, and long-term performance. Empirical studies confirm that stakeholder-oriented CSR improves customer retention, employee commitment, and innovation outcomes [24]. Thus, ST enables organizations to move beyond narrow financial indicators and assess broader impacts on sustainability. This framework makes CSR implementation more meaningful and contextually relevant, particularly in resource-constrained environments.

In SMEs, stakeholder relationships are often more directed, localized, and trust-based than in larger corporations. These firms operate closer to their communities and are more influenced by social expectations. Stakeholder Theory, therefore, offers a flexible framework for designing inclusive CSR policies that reflect local priorities. Stakeholders can be categorized into internal (e.g., employees, owners) and external groups (e.g., customers, suppliers, regulators, society) [25,26]. Understanding these categories helps SMEs tailor CSR strategies for maximum impact. Evidence suggests that aligning CSR initiatives with stakeholder needs improves both firm reputation and competitive positioning [27]. Moreover, stakeholder-oriented CSR can reduce conflict, increase transparency, and enhance strategic decision-making [28]. These insights are crucial for SMEs navigating institutional voids and regulatory weaknesses. By applying ST, firms can foster stronger stakeholder ties and develop CSR models that ensure long-term business sustainability. This theoretical foundation therefore supports our study’s aim to assess CSR’s influence on SME sustainability.

### 2.2 Conceptual framework and hypothesis development

**Fig 1:**
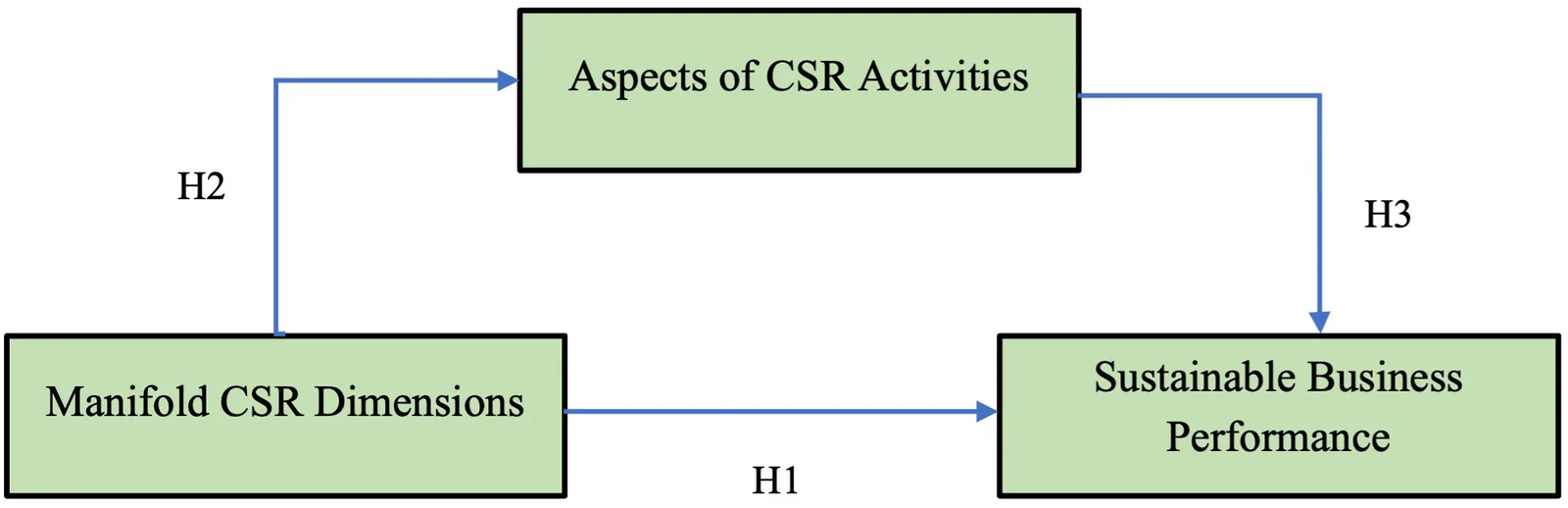
Conceptual research model.

Corporate social responsibility (CSR) includes several key dimensions: employee welfare, community engagement, customer responsibility, and environmental stewardship. Each dimension plays an equally important role in engaging diverse stakeholders, particularly within small and medium-sized enterprises (SMEs). In dynamic environments, SMEs must respond quickly to stakeholder expectations for achieving sustainable business performance (SBP). Clear and strategic CSR communication supports business objectives and enhances stakeholder trust and alignment. These CSR dimensions are deeply interrelated, increasing the complexity of managing corporate responsibilities effectively. Stakeholder Theory provides a valuable lens to understand how CSR dimensions contribute collectively to SBP [19,28]. Given SMEs’ embeddedness in complex stakeholder networks, coordinated communication is essential to sustain long-term relationships. An integrated CSR approach allows firms to communicate commitments clearly across all stakeholder groups [29]. Studies show that effective CSR implementation and communication enhance transparency, build reputation, and strengthen stakeholder loyalty. Fig 1 illustrates this conceptual relationship through the proposed research framework.

*This model depicts the hypothesized relationships among corporate social responsibility (CSR) constructs and business outcomes. H1 denotes the direct effect of Manifold CSR Dimensions on Sustainable Business Performance; H2 denotes the effect of Manifold CSR Dimensions on Aspects of CSR Activities; and H3 denotes the effect of Aspects of CSR Activities on Sustainable Business Performance. Arrows (◊) indicate hypothesized directional relationships.*

#### 2.2.1 Manifold CSR dimensions and sustainable business performance.

Corporate Social Responsibility (CSR) is a multidimensional construct comprising philanthropic, ethical, legal, and economic responsibilities. Carroll [30] conceptualized these four dimensions to demonstrate that businesses must balance profit with broader obligations. Philanthropy, though discretionary, is viewed as a strategic tool for enhancing corporate reputation and stakeholder trust [31,32]. When aligned with social expectations, philanthropic efforts promote goodwill and strengthen organizational legitimacy [33]. However, its financial value often depends on industry type and stakeholder perceptions [31,34]. Ethical CSR emphasizes fairness, transparency, and human rights, surpassing legal compliance to foster social trust [19,35]. Legitimacy Theory supports the idea that firms aligning with societal norms earn trust and long-term acceptance [36]. Yet, superficial or insincere ethical CSR initiatives may undermine credibility and stakeholder engagement [37]. Legal CSR focuses on adherence to labor, environmental, and consumer regulations to minimize risk and ensure accountability. According to Stakeholder and Institutional Theories, legal compliance builds legitimacy and reputation [28,38]. Nevertheless, an excessive focus on compliance may restrict innovation and increase costs [31]. Economic CSR underscores financial sustainability while addressing social and environmental concerns [31,39]. Research suggests it improves efficiency, market viability, and long-term profitability [40,41]. However, prioritizing short-term profits can undermine sustainable development goals [42]. Based on the findings, this study proposes and tests the following hypothesis to further examine the relationships:

***H1.*** *There is a considerable positive association between Carroll’s manifold CSR dimensions (including philanthropic, ethical, legal, and, economic) and sustainable business performance.*

#### 2.2.2 Manifold CSR dimensions and aspects of CSR.

Corporate Social Responsibility (CSR) enables firms to engage stakeholders across four key dimensions: employee, community, customer, and environment. Each dimension is vital to achieving sustainable business performance (SBP), particularly in stakeholder-sensitive sectors like small and medium-sized enterprises (SMEs). Employee-focused CSR emphasizes equity, inclusion, well-being, and professional development, which enhance satisfaction, retention, and innovation capacity. Transparent CSR communication builds trust, boosts employer branding, and strengthens stakeholder relationships [43,44]. Community-related CSR fosters goodwill through donations, sponsorships, and local development. These efforts enhance continuity and stakeholder support [11]. Such practices are especially valuable for SMEs operating within close-knit community structures. Firms that disclose community engagement demonstrate ethical commitment, which contributes to reputational capital and financial performance [45]. Customer-centric CSR includes ethical marketing, product safety, responsible sourcing, and honest communication. These efforts increase customer loyalty and brand equity [30,46]. Consumer behavior is influenced by clear labeling, sustainability, and ethical standards. Poorly executed CSR may damage trust, while effective practices build resilience and market differentiation [22]. Environmental CSR has become critical as firms face pressure to reduce ecological footprints. Sustainable practices promote long-term compliance and performance [47]. Proactive strategies reinforce legitimacy and stakeholder trust while advancing competitive differentiation [48]. Strategic CSR communication ensures alignment with stakeholder priorities, boosts engagement and sustains firm credibility [49,50]. Therefore, the study's findings form the basis for the following hypothesis:

***H2.*** *Manifold CSR dimensions have a considerable impact on strategic aspects of multi-stakeholder CSR activities (including environment, community, customer, and employee) in the communicating perspective of sustainable business performance of SMEs.*

#### 2.2.3 Aspects of CSR and sustainable business performance.

Corporate Social Responsibility (CSR) is essential for the long-term success of manufacturing SMEs in competitive markets. CSR balances stakeholder expectations while enhancing efficiency, regulatory compliance, and market reputation. Employee-focused CSR—such as ethical labor practices and workforce development—boosts job satisfaction, innovation, and employee retention [43,44]. CSR communication improves legitimacy, strengthens employer branding, and reduces turnover, resulting in better internal performance. Community-based CSR, including donations and local partnerships, enhances social cohesion and stakeholder relationships [11]. These initiatives foster business continuity and deepen local support networks. Transparency in community engagement builds corporate reputation and improves financial outcomes [45]. Customer-oriented CSR focuses on ethical practices, safe products, sustainability, and clear labelling [30,51]. These actions shape consumer trust, brand loyalty, and purchasing behaviour. Poorly executed CSR can damage brand image. Conversely, well-managed CSR fosters credibility and long-term customer relationships [22]. Environmental CSR is crucial for manufacturers, given their ecological footprint. Activities such as waste reduction and resource efficiency drive compliance and stakeholder trust [47]. Proactive environmental actions improve legitimacy and differentiate firms in sustainability-conscious markets [48]. Moreover, the attachment of CSR enhances stakeholder engagement and financial stability for SMEs [49]. Building on the above key insights, this study formulates the following hypothesis to guide further investigation:

***H3.*** *There is a significant impact of manufacturing SMEs' strategic aspects of CSR activities on sustainable business performance.*

## 3 Methodology

### 3.1 Instruments

This study employs a structured methodology to explore how various CSR dimensions and aspects affect sustainable business performance (SBP) in SMEs. Grounded in stakeholder theory, it adopts a multi-stakeholder communication perspective to assess CSR's strategic influence. The survey used closed-ended questions for clarity and ease of analysis. A five-point Likert scale, ranging from “strongly disagree” (1) to “strongly agree” (5), measured all responses. CSR is examined through two constructs: Dimensions of CSR (DCSR) and Aspects of CSR (ACSR). DCSR includes philanthropic, ethical, legal, and economic elements, adapted from Jing et al. [52], Masud and Hossain [53], and Turker [54]. ACSR captures stakeholder engagement across employees, customers, communities, and the environment [54]. SBP is assessed through profitability, market position, and operational efficiency [55]. All items were adapted from validated sources to ensure reliability and content validity. S1 Appendix presents the full list of measurement instruments used in the analysis.

### 3.2 Sample and data collection

This study targeted manufacturing SMEs in Bangladesh due to their central role in economic development and sustainability. Data were collected from Dhaka, Chittagong, and Barishal, which together represent 71% of SME activity [56,57]. A structured questionnaire was designed and pre-tested with academics and SME professionals to ensure clarity and relevance. A pilot study with 20 participants, conducted between January and February 2025 after the approval from the IRB (Institutional Review Board – Port City International University), confirmed its reliability. The final survey was distributed to 430 SME managers, producing 350 valid responses (81.40% response rate). Purposive sampling was used to select firms involved in sustainability initiatives like the Corporate Sustainability Index and UN Global Compact. Purposive sampling was appropriate for this study as it targeted SMEs actively engaged in CSR and sustainability initiatives. These firms were selected due to their relevance to the research objective, which required informed insights into CSR practices. The focus was not on generalizing to all SMEs but on analyzing CSR impact within sustainability-oriented firms. Thereafter, data were gathered through cross-sectional surveys and semi-structured interviews with SME stakeholders. Respondents included owners, managers, and employees. The sampling frame was developed using the BSMEF directory, focusing on 850 registered SMEs in six key sectors: textiles, food processing, jewelry, ceramics, carpets, and machine tools.

### 3.3 Data analysis tools and techniques

This study employed Partial Least Squares Structural Equation Modeling (PLS-SEM) to analyze relationships among CSR constructs and SBP. PLS-SEM is well-suited for exploratory research involving multiple constructs, non-normal data, and small to medium sample sizes [58]. It allows simultaneous testing of direct and indirect relationships, making it ideal for modeling multidimensional CSR. Unlike covariance-based SEM, PLS-SEM does not require data normality or large samples, offering greater flexibility [59]. This makes it appropriate for studies using formative and reflective constructs [60]. SmartPLS 3.2.9 software was used to test the measurement and structural models based on 350 valid responses.

To enhance explanatory power, this study integrates importance-performance map analysis (IPMA) within the PLS-SEM framework. IPMA identifies critical predictors by evaluating each construct’s relative importance (path coefficients) and performance (average latent scores) simultaneously. Importance was derived from the PLS path coefficients, while performance was computed from the average latent variable scores scaled from 0 to 100. IPMA provides practical guidance by problem-solving constructs with high importance but low performance, highlighting areas for managerial focus [61]. The use of IPMA aligns with stakeholder theory, emphasizing value creation through targeted CSR activities. IPMA extends traditional PLS-SEM findings by bridging statistical significance with actionable stakeholder relevance. This integration supports the study's goal to offer strategic insights for SMEs aiming to align CSR with sustainable business outcomes.

### 3.4 Multivariate assumptions

Before model validation, multivariate assumptions were assessed to ensure result reliability and statistical credibility [62]. *First,* normality was tested using skewness and kurtosis values, which were within ±2.58. Results confirmed that all variables were normally distributed and appropriate for parametric analysis [58]. *Second,* considering the issues of linearity, ANOVA’s deviation-from-linearity test and OLS regression were used to evaluate linear relationships. P-values supported the presence of significant linear patterns between predictors and outcomes. *Third,* scatterplots showed evenly dispersed residuals along the fitted line, confirming the assumption of homoscedasticity [63]. *Finally,* Harman’s single-factor test showed that one factor explained only 39.64% of the variance well below the 50% threshold [64]. Additional tests were applied due to criticisms of Harman’s test [65]. Inter-construct correlations were all below 0.90 [66], indicating no multicollinearity [67]. These results confirm the robustness and methodological soundness of the research model. Moreover, all tests validated the assumptions of normality, linearity, homoscedasticity, and CMB control.

## 4 Results

### 4.1 Demographic profile of the respondents

The demographic profile of the respondents (n = 350) reveals the representation across managerial and operational roles. Managers constituted the largest group at 51.7%, followed by employees (28%) and business owners (20.3%). This mix ensures practical insights into SME operations and strategic perspectives. The majority of SMEs surveyed were sole proprietorships (91.1%), while 8.9% operated under partnership arrangements. Sector-wise, electronics accounted for the largest share (39.1%), followed by pharmaceuticals (22.3%), transport (16%), construction (13.1%), and textiles (9.4%). This sectoral composition emphasizes the dominance of manufacturing and technology-based SMEs. In terms of market reach, 53.7% of firms served the national market, 28.9% engaged in exports, and 17.4% operated locally. Regarding business maturity, 50.6% had operated between 5–9 years, 30.9% exceeded 10 years, and 18.6% were categorized as start-ups. These demographics suggest a sample composed mainly of established SMEs, offering grounded insights into CSR practices across business lifecycles. A detailed breakdown of these characteristics is provided in Table 1.

**Table 1:** Demographic profile of the respondents (n = 350).

SMEs Profile	Category	Frequency	Percentage	Cumulative
Types of Respondents	Owner	71	20.3	20.3
	Manager	181	51.7	72.0
	Employee	98	28.0	100.0
Types of Ownership	Sole Proprietorship	319	91.1	91.1
	Partnership	31	8.9	100.0
Type of Industry	Textile	33	9.4	9.4
	Construction	46	13.1	22.6
	Pharmaceuticals	78	22.3	44.9
	Electronics	137	39.1	84.0
	Transport	56	16.0	100.0
Types of market served	Local	61	17.4	17.4
	National	188	53.7	71.1
	Export	101	28.9	100.0
Year in Business	Less than 5 years	65	18.6	18.6
	More than 5–9 years	177	50.6	69.1
	Above 10 years	108	30.9	100.0

### 4.2 Findings from the symmetrical pre-hoc analysis (PLS-SEM)

#### 4.2.1 Assessment of the measurement model.

The measurement model was assessed through construct reliability and convergent validity, following established standards, as depicted in Table 2. Cronbach’s alpha (α) and composite reliability (CR) both exceeded the 0.70 threshold, confirming strong internal consistency [68]. Values ranged from 0.891 to 0.938 for α and 0.768 to 0.894 for CR. Convergent validity was established using factor loadings (λ) and average variance extracted (AVE). All factor loadings exceeded 0.70, while AVE values were above 0.50, satisfying Hair et al.’s [58] criteria. Thus, the constructs demonstrated robust reliability and convergent validity, supporting the truthfulness of the measurement model.

**Table 2:** Convergent validity and reliability assessment.

Construct	α	rho_A	CR	AVE
Manifold CSR dimensions	0.891	0.895	0.768	0.667
Aspects of CSR	0.933	0.936	0.894	0.681
Sustainable business performance	0.938	0.939	0.891	0.844

Furthermore, discriminant validity was assessed using the Fornell-Larcker criterion and the Heterotrait-Monotrait (HTMT) ratio. HTMT values were all below the 0.85 thresholds, confirming discriminant validity [69]. Additionally, AVE square roots exceeded inter-construct correlations, satisfying Fornell and Larcker’s [70] criterion. As shown in Table 3, both tests supported the model’s discriminant validity. Consequently, the measurement model was thoroughly validated and confirmed to be both reliable and distinct across constructs.

**Table 3:** Discriminant Validity Analysis.

Fornell-Larcker’s Criterion				Heterotrait-Monotrait Ratio (HTMT)			
	CSRD	CSRA	SBP		CSRD	CSRA	SBP
CSRD	0.752	---	---	CSRD	---	---	---
CSRA	0.494	0.826	---	CSRA	0.534	---	---
SBP	0.459	0.370	0.919	SBP	0.498	0.392	---

**Note:** Manifold CSR dimensions (CSRD); aspects of CSR (ACSR); sustainable business performance (SBP).

#### 4.2.2 Assessment of the structural model.

After validating the measurement model, the structural model was assessed to examine inter-construct relationships. Path coefficients (β), t-statistics, and p-values were used to evaluate each hypothesized relationship. Table 4 presents the PLS-SEM results, confirming statistically significant relationships among the variables. CSRD significantly influenced SBP with β = 0.365, t = 2.949, and p < 0.05. CSRD also significantly predicted ACSR with β = 0.494, t = 4.231, and p < 0.05. Likewise, ACSR showed a significant positive impact on SBP with β = 0.189, t = 3.039, and p < 0.05. These results confirm that hypotheses H1, H2, and H3 are supported. Thus, CSR dimensions and aspects collectively contribute to improved business sustainability. The findings reinforce the strategic value of CSR in enhancing stakeholder trust and organizational performance in SMEs.

**Table 4:** Structural Model.

Hypothesis	Path	β	t-statistics	Comments
H1	CSRD → SBP	0.365	2.949	Supported
H2	CSRD → ACSR	0.494	4.231	Supported
H3	ACSR → SBP	0.189	3.039	Supported

**Note:** Manifold CSR dimensions (CSRD); aspects of CSR (ACSR); sustainable business performance (SBP).

#### 4.2.3 Explanatory power of model fit.

To assess the structural model, R^2^ was used to determine the variance explained by independent variables, as depicted in Table 5. R^2^ values of 0.244 for ACSR and 0.238 for SBP indicate moderate explanatory power [71]. These results suggest the model explains 24.4% and 23.8% of the variance in ACSR and SBP respectively. The Stone-Geisser Q^2^ test was also performed to evaluate the predictive relevance of endogenous variables. Q^2^ values were 0.429 for ACSR and 0.371 for SBP, both exceeding the minimum threshold of zero. This confirms that the model holds strong predictive power for key constructs within the framework. Effect size (f^2^) was used to examine the impact of each exogenous variable on the endogenous variables. According to Cohen’s [72] criteria, f^2^ values of 0.370 (ACSR), and 0.365 (SBP) are large. These findings support the strong influence of CSR constructs on business performance in stakeholder-driven SMEs. Hence, the model is both explanatory and predictive in evaluating CSR’s strategic role in sustainable performance.

**Table 5:** Explanatory power.

Construct	R-Square (R^2^)	Q-Square (Q^2^)	F-Square (f^2^)
Aspects of CSR	0.244	0.429	0.370
Sustainable business performance	0.238	0.371	0.365

### 4.3 Findings from the symmetrical post-hoc analysis (IPMA)

This study integrated importance-performance map analysis (IPMA) into the PLS-SEM framework to assess key CSR predictors of SBP. IPMA offers a broader evaluation by combining path coefficient importance and performance metrics of latent constructs [61]. It reveals which constructs deserve strategic attention based on both their contribution and execution performance within the model. The analysis focused on two core CSR variables: corporate social responsibility dimensions (CSRD) and aspects of CSR (ACSR). As reported in Table 6, ACSR showed the highest importance (0.137) but lower performance (54.976). Conversely, CSRD displayed the highest performance (58.640) with strong importance, positioning it as a well-executed and influential predictor. This performance-importance gap in ACSR suggests a need for enhanced implementation strategies aligned with stakeholder communication and expectations.

**Table 6:** IPMA for performance impact.

Latent Constructs	Importance	Performance
Manifold CSR dimensions	0.122	58.640
Aspects of CSR	0.137	54.976

Moreover, managers should therefore refine CSR activities by improving delivery effectiveness and stakeholder-centric design across all dimensions. High-performing CSRD indicates mature discovery systems, yet continuous improvement in transparency and engagement will strengthen CSR’s strategic role. CSR should not be a symbolic practice but integrated into core values for authentic, measurable, and strategic advantage. The strategic alignment of CSR goals with stakeholder priorities creates mutual value and long-term sustainability for SMEs. By utilizing IPMA, firms can optimize resource allocation to improve underperforming but high-impact CSR areas. This enhances overall sustainable business performance by ensuring CSR practices are both meaningful and effectively operationalized. Thus, IPMA results confirm CSRD and ACSR as pivotal contributors to business resilience.

## 5 Discussions and implications

### 5.1 Discussions

The study provides strong empirical support for Carroll’s multidimensional CSR framework namely economic, legal, ethical, and philanthropic as foundational to SBP. Findings confirm a significant positive relationship between CSR dimensions and SBP, thereby validating H1 of the study. Philanthropic CSR contributes by building goodwill, enhancing corporate image, and reinforcing stakeholder engagement through social trust mechanisms. Ethical CSR strengthens transparency, governance, and moral responsibility, creating a favorable perception and long-term stakeholder loyalty [73]. The legal dimension is reframed as a strategic enabler, supporting accountability and reducing compliance-related risks. Economic CSR emerged as the most influential factor for SBP, emphasizing ethical profits and sustainable economic development locally. These dimensions, taken together, indicate that CSR is not a cost burden but a strategic investment for SMEs. Thus, CSR should be operationalized systemically and strategically to improve stakeholder trust and maintain sustainable growth across business functions.

Likewise, this study adds depth to CSR literature by emphasizing its stakeholder-oriented communication aspect, validated through H2, and H3. The findings reveal that environmental CSR significantly influences SBP through better compliance, environmental stewardship, and corporate legitimacy [74]. CSR efforts directed at community well-being build social capital and foster long-term relationships, increasing social license to operate. Customer-focused CSR enhances satisfaction and loyalty by promoting ethical practices, transparency, and sustainability in value chains [75]. Employee-centered CSR boosts morale, productivity, and innovation by ensuring fair treatment, inclusion, and work-life balance [76]. These integrated CSR practices demonstrate how SMEs can achieve superior SBP by aligning social expectations with business strategies. Stakeholder theory underpins these relationships, advocating for multi-directional communication and inclusive decision-making in CSR design and execution. Therefore, CSR should be embedded within core business functions to support balanced economic, social, and environmental sustainability objectives.

Additionally, post-hoc IPMA results highlight the importance and performance of key CSR constructs in predicting SBP outcomes. CSRD showed the highest performance, indicating effective integration into operational strategy and stakeholder engagement mechanisms. Meanwhile, ACSR demonstrated the highest importance, yet exhibited slightly lower performance compared to CSRD. This gap signals a significant opportunity for SMEs to optimize CSR activities and enhance communication for greater impact. Effective CSR aspects enhance legitimacy, while poorly executed CSR diminishes perceived authenticity and long-term stakeholder trust. IPMA confirms that CSR, when well-communicated and aligned with expectations, amplifies stakeholder commitment. Thus, the dual consideration of importance and performance is essential for prioritizing CSR actions in resource-constrained SMEs. Both CSRD and ACSR significantly influence SBP, suggesting that SMEs must strategically manage both dimensions to gain sustainable advantage.

### 5.2 Theoretical implications

This study makes a significant theoretical contribution by advancing the application of stakeholder theory (ST) within CSR research. Stakeholder theory positions CSR as a strategic mechanism to address diverse stakeholder interests across operational contexts. Our findings affirm that stakeholder-oriented CSR directly improves business sustainability, particularly in resource-constrained SME environments. Internal CSR practices, like employee development, enhance job satisfaction, loyalty, and productivity within manufacturing SMEs [22]. CSR focused on customers strengthens brand equity through ethical marketing, transparency, and customer-centric innovations [23]. Community engagement builds trust, which contributes to social goodwill and reputation management over the long term. These insights reinforce that CSR's effectiveness depends on how closely strategies are aligned with stakeholder needs. ST, therefore, helps frame CSR as a proactive investment rather than a compliance tool. The theory provides a robust foundation for understanding CSR's integration into long-term sustainable performance in emerging markets.

This study contributes theoretically by validating Carroll’s CSR Pyramid in the SME context. The findings also extend Carroll’s insight by demonstrating the interaction between CSR aspects and strategic business outcomes. While Carroll saw philanthropy as discretionary, our results show it holds practical value for stakeholder trust-building. Ethical and environmental CSR were strongly tied to brand reputation, customer loyalty, and market positioning. This suggests CSR in SMEs is no longer optional but essential for adaptive capability and stakeholder responsiveness. Unlike Carroll’s linear hierarchy, SMEs integrate these dimensions dynamically based on stakeholder needs and resource availability. Therefore, this study offers a contextual reinterpretation of Carroll’s model within multi-stakeholder, performance-driven SME environments. It confirms CSR’s strategic integration is necessary for sustainability in socially and institutionally demanding markets.

### 5.3 Managerial implications

This study offers strategic guidance for SME managers aiming to align CSR with sustainable business performance. CSR should be regarded not merely as a compliance obligation but as a strategic enabler for long-term growth. Managers must prioritize value-driven CSR initiatives that address stakeholder expectations while meeting institutional requirements. Adapting CSR activities to local social and cultural contexts enhances organizational legitimacy and improves stakeholder engagement. A relational approach to CSR shaped by continuous stakeholder feedback can refine program design and boost responsiveness. Embedding CSR within long-term strategic planning strengthens operational resilience, enhances competitive advantage, and supports inclusive growth. These insights also carry important implications for policymakers. Targeted regulatory frameworks and institutional support are essential to encourage CSR adoption in resource-constrained sectors like SMEs. Firms should integrate CSR within their corporate governance structures, aligning economic, legal, ethical, and discretionary responsibilities. Philanthropic initiatives, such as health, education, or community development programs, should be strategically linked to business objectives. This integrated CSR approach promotes organizational stability, deepens stakeholder trust, and contributes meaningfully to social and environmental sustainability.

## 6 Conclusion, limitations, and future research directions

This study examined the relationships between CSR dimensions-and-aspects aligned with sustainable business performance in Bangladeshi manufacturing SMEs. Guided by stakeholder theory and a multi-stakeholder communication lens, we applied PLS-SEM and IPMA to validate key paths. Both pre-hoc and post-hoc analyses confirmed that manifold CSR dimensions and activities significantly influence SBP outcomes. Notably, practices like recycling and renewable energy adoption emerged as impactful strategies supporting sustainability performance. The study supports prior evidence that internal CSR such as ethical governance and employee engagement and external CSR like community programs and environmental initiatives create a synergistic sustainability effect. Strategic integration of CSR with regulatory collaboration enhances the growth potential of SMEs in dynamic markets. Findings also reveal that integrating CSR goals produces stronger performance outcomes for SMEs. Each CSR dimension must align with its related activities to yield maximum impact. Finally, it emphasizes the need for collaborative stakeholder engagement. Such cooperation ensures that CSR meaningfully addresses SMEs’ sustainability imperatives across evolving institutional and market landscapes.

Despite valuable contributions, this study has several limitations that open pathways for future research. Its geographical focus on three Bangladeshi cities restricts broader generalization across other regions. Future studies should incorporate larger and more diverse SME samples across sectors. A longitudinal design could reveal changes in CSR strategies over time. Political influences on CSR disclosure in government-backed SMEs deserve further investigation. Including mediating and moderating variables may strengthen causal inferences. Expanding the research into service industries would also enhance generalizability. This study offers theoretical and practical insights, encouraging SMEs to embrace CSR for sustained growth and stakeholder trust.

## Supporting information

S1 Appendix List of measurement items. (DOCX)
