# Supplementary material for: Rationalizing manifold CSR dimensions-and-aspects towards sustainable business performance of small and medium-sized enterprises: Findings from pre-hoc and post-hoc analysis
Source: PLoS One. 2026 Sep 15;21(9):e0353992. doi: 10.1371/journal.pone.0353992 (PMC13577511; doi:10.1371/journal.pone.0353992)
Supplement: Supplementary file 1 [file pone.0353992.s001.docx]

**Supporting Information File**

**S1 Appendix.** List of measurement items

| Manifold CSR dimensions | Loading (λ) |
| --- | --- |
| CSRD1: Our organization consistently allocates sufficient financial resources to support charitable causes. | **0.798** |
| CSRD2: We place a strong emphasis on fulfilling our social responsibilities to society at large. | **0.845** |
| CSRD3: We engage in market competition by adhering to strict ethical standards and values. | **0.824** |
| CSRD4: We proactively avoid all forms of unethical or unfair competitive behavior. | **0.869** |
| CSRD5: We comply fully and promptly with all relevant legal frameworks and regulations. | **0.835** |
| CSRD6: Legal compliance is upheld across all our organizational operations and decisions. | **0.837** |
| CSRD7: We strive to generate employment opportunities through our strategic initiatives. | **0.852** |
| CSRD8: Our long-term growth strategy includes a strong commitment to future generations and sustainability. | **0.845** |
| Aspects of CSR activities | |
| ACSR1: We design and manufacture products with a focus on environmental friendliness and safety. | **0.806** |
| ACSR2: We implement formal environmental policies to minimize ecological impact during operations. | **0.788** |
| ACSR3: We support local scholarships and community-based research through dedicated funding initiatives. | **0.873** |
| ACSR4: We engage in community development projects such as building educational and housing infrastructure. | **0.770** |
| ACSR5: Our products and services are developed to ensure safety for people and the natural environment. | **0.781** |
| ACSR6: We provide honest advertising and ensure product safety, durability, and functional reliability. | **0.721** |
| ACSR7: We offer performance-based incentives and bonuses to foster employee motivation and competitiveness. | **0.740** |
| ACSR8: We actively involve employees in participatory decision-making processes. | **0.750** |
| Sustainable business performance | |
| SBP1: CSR integration supports the long-term sustainability of both society and the business itself. | **0.911** |
| SBP2: CSR initiatives enhance employee loyalty, motivation, commitment, and overall organizational efficiency. | **0.928** |
| SBP3: CSR improves our corporate image and strengthens relationships with all key stakeholders. | **0.927** |
| SBP4: CSR contributes directly to the operational efficiency required for sustainable business success. | **0.909** |
